# Ergot

**Published:** 1854-10

**Authors:** Paul Dubois


Ergot: Circumstances which contra-indicate its use during labor; by M. Paul Dubois, M. D.—Ergot, besides its well known hemostatic properties, is useful when, from inertia, the uterine contractions are insufficient to complete delivery. It is, however, to be regreted that this valuable remedy is so often improperly used. We have frequently known it given when it was entirely unavailing, because unsuited to the case. This abuse, and the sad effects consequent upon it, have contributed very much to discredit ergot in the minds of many practitioners. We should, nevertheless, not be deprived of so precious an agent from the mere fact that it has been injudiciously administered. We should rather endeavor to ascertain the circumstances under which it may be advantageously prescribed, and distinguish these from the contraindications to its use. The circumstances under which the uterine contractions may be impaired without calling for the use of ergot are sufficiently numerous to merit attention. The following are some of the most common:

1st. Debility dependent upon a natural state, or induced by previous disease. This requires tonics, broth, wine, &c., for the purpose of improving the strength of the patient during labor.

2d. Extreme distension of the uterus from an excessive quantity of liquor amnii, which induces incomplete paralysis. The uterine fibers being inordinately distended, the membranes should be punctured whenever the os tincæ is one-fourth dilated, or about the size of a half dollar.

3d. Congestion of the face, attended with impaired contractions from plethora. The patient should then be bled.

4th. Mental disturbance from whatever cause, sometimes from the presence of particular persons in the chamber, may suspend the uterine contractions. The woman must be quieted and the causes of annoyance removed.

5th. Great elevation of temperature in the apartment may lessen the contractions by the cerebral congestion it induces. The room should, in such cases, be well ventilated and cooled if possible.

6th. Pain other than that of urine. Retention of urine, may be so painful as to impair the contractions; catheterism should then be practiced. Very great pain in the loins may likewise affect them, in which case we should apply the forceps if labor is sufficiently advanced; if not, wait patiently. The same may be said with regard to the headache which sometimes attends each abdominal contraction.

7th. The premature discharge of the waters makes labor slow, because, like all hollow organs, the uterus contracts most forcibly upon unyielding contents, and that when the membranes are ruptured the descent of the fœtus at each contraction lessens its force.

8th. The uterine contractility may become exhausted by the tardy rupture of the membranes in consequence of the firmness of these; rupture them. Rigidity of the os tincæ from phlogosis may do the same; resort to belladonna or to general bleeding. If the cause be unyielding induration of the tissues of the cervix uteri, of which we have recently seen a case, the knife should relieve the resistance.

9th. There may be posterior obliquity of the cervix which will lessen the force of the contractions. If the os does not dilate of itself (and cannot be rectified by the finger—Ed.) we may have to divide its anterior lip with a probe pointed bistoury.—*Southern Med. & Sur. Journal.*

